# Ligand-gated ion channel interacting proteins and their role in neuroprotection

**DOI:** 10.3389/fncel.2014.00125

**Published:** 2014-05-09

**Authors:** Shupeng Li, Albert H. C. Wong, Fang Liu

**Affiliations:** Campbell Family Mental Health Research Institute, Centre for Addiction and Mental Health, University of TorontoToronto, ON, Canada

**Keywords:** ligand-gated ion channels, protein-protein interaction, receptor complex, glutamate receptors, dopamine receptors

## Abstract

Ion channel receptors are a vital component of nervous system signaling, allowing rapid and direct conversion of a chemical neurotransmitter message to an electrical current. In recent decades, it has become apparent that ionotropic receptors are regulated by protein-protein interactions with other ion channels, G-protein coupled receptors and intracellular proteins. These other proteins can also be modulated by these interactions with ion channel receptors. This bidirectional functional cross-talk is important for critical cellular functions such as excitotoxicity in pathological and disease states like stroke, and for the basic dynamics of activity-dependent synaptic plasticity. Protein interactions with ion channel receptors can therefore increase the computational capacity of neuronal signaling cascades and also represent a novel target for therapeutic intervention in neuropsychiatric disease. This review will highlight some examples of ion channel receptor interactions and their potential clinical utility for neuroprotection.

Efficient neurotransmission requires the precise interplay of various neurotransmitter receptors at pre- and post-synaptic compartments. Ligand-gated ion channels play a central role in intercellular communication in the nervous system. Ion channels are the cellular machinery for ion flux across the membrane and therefore the basis of electrical excitation of neurons. Ligand-gated ion channels are oligomeric protein assemblies that convert a chemical signal into an ion flux through the post-synaptic membrane, and are involved in basic brain functions such as attention, learning, and memory (Ashcroft, 2006). This paper will review some interactions between ionotropic receptors and other proteins that affect signaling, metabolism, and trafficking. We will detail some of the interactions between G-protein coupled receptors and ion channels, ion channels with intracellular proteins, and ion channels with other ion channels. Specifically, we will discuss how these receptor-protein interactions may help to understand pathology of the nervous system, and offer the hope of new treatment targets for neuropsychiatric disease.

Most eukaryotic membrane channels are composed of several subunits. Pentameric ligand-gated ion channels have five subunits arranged in a pseudosymmetric rosette around the centrally located ion channel pore. Each subunit consists of an extracellular domain that contains the ligand-binding site and a transmembrane ion-pore domain (Kozuska and Paulsen, 2012). The agonist binding site is located at the boundary between subunits in the extracellular domain whereas the ion pore is formed by the transmembrane helices M2, which differentiate axial cationic and anionic channels (Dacosta and Baenziger, 2013).

The modular construction of ionotropic receptors increases the diversity of ligand binding and ion selectivity through the mixing and matching of various subunits (Rojas and Dingledine, 2013). Various other proteins are involved in controlling the type and position of particular subunits in a given channel, as well as the function of the function of the assembled channel. Thus, the function of many receptor proteins can only be fully understood in the context of their interactions with other proteins, the “interactome” (Li et al., 2013a). Through this extensive protein network, the binding of agonist to an ionotropic receptor can exert effects beyond simple current flow, including gene transcription, cytoskeletal rearrangement, and protein synthesis, degradation and transport.

The ligand-gated ion channel superfamily includes nicotinic acetylcholine receptors (nAChRs), adenosine triphosphate (ATP) receptors, γ-aminobutyric acid (GABA), glutamate, glycine, and 5-hydroxytryptamine (5-HT) receptors (Dent, 2010). The “Cys-loop” sub-family includes the ionotropic receptors for nicotine, GABA, glycine, and 5-HT. “Pore-loop” channels include inwardly-rectifying potassium channels and the glutamate receptors of the AMPA (α-amino-3-hydroxy-5-methyl-4-isoxazolepropionic acid) subtype and NMDA (N-methyl-D-aspartate) receptors (Connolly and Wafford, 2004). The structure and basic features of several important ion channel types is briefly reviewed below to provide some background, before discussing ion channel interactions with other proteins.

AMPA receptors are the major mediator of fast excitatory synaptic transmission in the mammalian central nervous system. Most of these ionotropic glutamate receptors are permeable to calcium and other cations, thus facilitating depolarization of the cell membrane. AMPA receptors contain four subunits, consisting of two dimers formed by combinations of four pore-forming subunits GluA 1–4. GluA 1/2, and GluA 2/3 are the predominant AMPA receptor types (Anggono and Huganir, 2012). Each subunit is an integral membrane protein with an extracellular amino terminal domain, three membrane-spanning domains (M1, M3, and M4), a hydrophobic hairpin domain (M2) that forms the channel pore, and three intracellular domains: Loop1, Loop2, and the carboxyl tail. Although the carboxyl tail is crucial for receptor trafficking and function, other intracellular domains also play an important role in the regulation of AMPA receptor trafficking (Rojas and Dingledine, 2013). The pore lining domain also influences the AMPA receptor trafficking, early in the secretory pathway.

NMDA receptors are also ionotropic glutamate receptors permeable to calcium and sodium, and are critical for synaptic plasticity, learning, and memory. These receptors are involved nicotine addiction and neurological disorders such as ischemic stroke (Dingledine et al., 1999; Albensi, 2007). NMDA channel opening is gated by several elements, including the binding of glutamate to the NR2 subunit, a co-ligand glycine which binds to a separate site on the NR1 subunit, and the removal of a magnesium ion block by depolarization. Thus NMDA channels require both agonist stimulation and depolarization of the post-synaptic membrane for current flow, effectively detecting coincident pre- and post-synaptic activation. Like AMPA receptors, NMDA receptors consist of four subunits, two of which are obligatory GluN1 and two which are modulatory GluN2. These subunits are present in various isoforms that in the NR1 subunit are generated by alternative splicing of the GRIN1 gene. The GluN2 subunits are each encoded by different genes and the subunits are named: NR2A, B, C, and D (Durand et al., 1992; Sugihara et al., 1992; Hollmann et al., 1993; Michaelis, 1998). The exact subunit composition of native NMDARs remains unknown, but it has been suggested that native NMDARs in rat hippocampus largely contain NR1/2A subunits. The intracellular C-terminal (Allison et al., 1998) contains potential sites of protein phosphorylation by different kinases, and motifs for interacting with various protein partners.

For pentameric ligand-gated ion channels such as the GABA_A_ receptor, each subunit contains an extensive N-terminal extracellular domain and a short extracellular C-terminal sequence containing the ligand binding sites. Structurally, each receptor is composed of four closely-spaced transmembrane domains including the ion channel wall primarily in M2, and a variable length cytoplasmic loop between M3 and M4. With both the N-terminus and C-terminus of receptor subunits extending outside the cell membrane, the intracellular M3-M4 loop becomes the most important domain interacting with the intracellular environment. The M3-M4 intracellular loop (IL2) contains protein-protein interaction domains involved in regulating synaptic localization and intracellular trafficking (Maksay, 2009). Despite being highly variable in length and amino acid sequence, the IL2 loop usually contains numerous regulatory motifs and binding sites that differ in each subunit isoform. Moreover, this IL2 loop usually contains sites for phosphorylation, palmitoylation, ubiquitination and other modifications that modulate protein interactions and eventually determine the clustering, stability, and function of ligand-gated ion channels.

Within the central nervous system (CNS), nicotine acts on nicotinic receptors (nAChRs) to initiate various physiological and pathological processes at the cellular and circuit level. Similar to the NMDAR, nAChR is also a ligand-gated ion channel receptor with high Ca^2+^ permeability. The pentameric nAChR has a number of different subtypes, each with individual pharmacological and physiological profiles and a distinct anatomical distribution in the brain (Gotti et al., 2009). There are 11 neuronal nAChR subunits identified in mammals, including eight α(α2 –α9) and three β (β 2– β 4). Unlike NMDAR, nAChR can exist as both hetero-metric and homo-metric-assemblies of these subunits (Albuquerque et al., 2009). In rodents, the β 2 subunit is found throughout the CNS, mostly as part of the α4β 2 heteromeric receptor, which exhibits high affinity for nicotine. The second common AChR isoform is the α7 homopentamer, expressed mainly in the cortex, hippocampus, and limbic areas.

Numerous proteins are known to bind to the different subunits of pentameric ligand-gated ion channel receptors at specific subcellular localizations. These interacting proteins can both modulate ion channel function and influence the localization of the channel in the cell and in the membrane. There are several major categories of proteins that ligand-gated ion channels interact with: G-protein coupled receptors, intracellular proteins, and other ligand-gated ion channels (Kittler and Moss, 2003; Li et al., 2012; Rojas and Dingledine, 2013). We will discuss each of these types of interaction with examples drawn from our previous work.

## Ligand-gated ion channel interactions with G-protein coupled receptors

There are several ion channel interactions with G-protein coupled receptors reported by our group, all involving dopamine receptors: D5-GABA_A_ (Liu et al., 2000), D1-NMDA (Lee et al., 2002; Pei et al., 2004), and D2-AMPA (Zou et al., 2005).

First discovered in 2000, the interaction between γ-aminobutyric acid A receptors and the dopamine D5 receptor was one of the earliest examples of interactions between metabotropic and ionotropic receptors (Liu et al., 2000). The GABA_A_ receptor is the major inhibitory neurotransmitter receptor that conducts chloride ions, resulting in hyperpolarization of the cell membrane and thus inhibiting neuron depolarization. The second intracellular loop of the GABA_A_ γ2 (short) receptor subunit binds directly to the carboxy-terminus of the D5 receptors. This interaction reduces D5 receptor-mediated cAMP accumulation without altering affinity of the D5 for endogenous and experimental ligands. Conversely, activation of D5 receptors reduces GABA_A_ currents and this is not dependent on PKC or PKA. Because these two receptors co-localize in hippocampal and cortical neurons, the bi-directional modulation is potentially important for a number of behaviors and brain disorders. A notable feature of this reciprocal modulation is that it is independent of the classical signaling pathways for each individual receptor.

The interaction between the D1 and NMDA receptor involves direct binding between the carboxy termini of the D1 receptor and the NR1-1a and NR2A subunits of the NMDA receptor (Lee et al., 2002). Because there are two interacting components in the NMDA receptor, the functional consequences are more complicated than with the D5-GABA_A_ interaction. Although the D1-NMDA interaction occurs in the absence of D1 receptor agonist stimulation, the D1 agonist SKF 81297 decreases the amount of D1-NMDA complex. However, the agonist affects only D1-NR1-1a binding, not the D1-NR2A interaction.

Similar to the D5-GABA_A_ interaction, D1 agonist stimulation reduces NMDA currents as well as reducing the slope of the current-voltage curve without altering the reversal potential. This decrease in NMDA conductance is accompanied by a reduction in NMDA cell surface expression. It is independent of PKA/PKC pathways and is mediated by the D1 interaction with the NR2A subunit. The D1-NR1-1a interaction mediates neuroprotective effects of D1 receptor activation, and these effects are not dependent on G-protein signaling but rather are dependent on a PI-3-kinase mechanism. The proposed model is that agonist activation of D1 receptors promotes dissociation of the D1-NR1-1a interaction, allowing the NR1-1a carboxy tail to bind CaM and PI-3 kinase, thereby initiating protective cellular cascades. Conversely, the D1 receptor is also regulated by NMDA receptor activation, which increases cell surface D1 receptor expression, and enhances cAMP accumulation in a SNARE-dependent fashion (Pei et al., 2004).

Dopamine D1 receptors also have a direct physical interaction with N-type calcium channels (Kisilevsky et al., 2008). In prefrontal cortex neurons dopamine D1 receptor activation reduced calcium influx through voltage-gated calcium channels, likely through both voltage-dependent G-protein-mediated pathways and through PKA-mediated pathways. The D1-Cav2.2 channel interaction also regulates cell surface expression and distribution of the channel, allowing effective dopaminergic regulation of backpropogating action potentials that are mediated by N-type calcium channels.

## Ligand-gated ion channel interactions with intracellular proteins

The second category of ion channel interactions to be considered is with intracellular proteins. For the GABAA receptor, multiple interacting proteins have been characterized, that play a role in cytoskeletal coupling by gephyrin, radixin, and F-actin and in signaling modulation by RAFT1 and collybistin (Chen and Olsen, 2007). The functional characteristics of this modulation are related to their subcellular localization with receptors. For example, GABARAP, GODZ, and Plic1 interact with the intracellular parts of the GABAA receptor, while GRIP1 and gephyrin are localized to sub-membrane and intracellular compartments.

Our group has recently reported an interaction between the N-terminus of the GluR2 sub-unit of the AMPA receptor and glyceraldehyde-3-phosphate dehydrogenase (GAPDH) (Wang et al., 2012; Zhai et al., 2013). GAPDH is a metabolic enzyme that participates in glycolysis and also plays a role in apoptosis (Tarze et al., 2007). AMPA receptor activation by agonist increases formation of the GluR2-GAPDH complex and promotes cellular internalization. Blocking this interaction with a peptide protects cells against glutamatergic excitotoxicity and ischemia-induced neuronal damage. This interaction is therefore of potential clinical interest for the treatment of ischemic stroke or other conditions with neuronal damage.

Another ion channel-protein interaction that has been of interest because of neuroprotective effects is between the PDZ domain of post-synaptic density 95 (PSD95) protein and carboxyl terminus of the NMDA NR2 subunit (Kornau et al., 1995; Niethammer et al., 1996; Cui et al., 2007). This interaction couples NMDA receptor activation to nitric oxide neurotoxicity (Sattler et al., 1999), and disrupting this interaction can protect neurons from excitotoxicity and ischemic brain damage both *in vitro* and *in vivo* (Aarts et al., 2002). PSD-95 also interacts with and suppresses the tyrosine kinase Src and attenuates Src-mediated NMDA receptor upregulation (Kalia et al., 2006). Consistent with these findings, inhibitors of PSD-95 also show neuroprotective effects in animal models of stroke (Sun et al., 2008).

While several examples of direct interactions between ion channels and G-protein coupled receptors have been discussed above, these two types of receptors can also exert functional crosstalk through indirect interactions. For example, the presynaptic voltage-gated calcium channels that influence neurotransmitter release are regulated by G-protein activation and protein kinase C-dependent phosphorylation through binding to Gβγ (Zamponi et al., 1997). G-protein modulation of N-type calcium channels also involves syntaxin 1A, a member of the SNARE protein complex responsible for synaptic vesicle fusion during neurotransmitter release (Jarvis et al., 2000). An additional modulator is cysteine string protein or CSP, which also bind to N-type calcium channels in conjunction with G-proteins to exert a tonic inhibition of the channel (Magga et al., 2000). In the case of G-protein activation in inwardly-rectifying potassium channels (GIRK), the Gβγ directly gates ion channel opening by binding to the intracellular pore of the channel (Nishida and MacKinnon, 2002).

## Ligand-gated ion channel interactions with other ion channels

Ion channel receptors can also interact with other ion channels, such as the interaction between the α7 nicotinic acetylcholine receptors and NMDA receptors (α7nAChR-NMDA) (Li et al., 2012, 2013b). The carboxy tail of the NMDA receptor NR2 subunit binds directly with the second intracellular loop of the α7nACh receptor, and the interaction promotes ERK1/2 phosphorylation. This interaction is of clinical interest since nicotine increases formation of the complex, and disrupting the α7nAChR-NMDA interaction blocks cue-induced reinstatement of nicotine self-administration in the rat. This behavioral test is a model of relapse in nicotine addiction, suggesting that the α7nAChR-NMDA interaction could be a useful target for novel smoking cessation therapies.

## Targeting ligand-gated ion channel interactions for neuroprotection

Because of the involvement of ion channel receptors in neuronal death from excitatory glutamate stimulation, there has been considerable interest in these receptors as therapeutic targets for the treatment of brain disorders involving neuronal death, such as ischemic stroke. Ischemic stroke is a major medical problem that affects millions of people world-wide. Current acute post-stroke treatment is focused on lysing the clot obstructing arterial blood flow by using a tissue plasminogen-activator. Due to a very short time window for effectiveness and the potential for intracranial bleeding, few patients can benefit from this treatment (Grossman and Broderick, 2013). Therefore, there is a major need for new and safer drugs that can reduce the extent of brain injury from ischemic stroke.

An alternative strategy for post-stroke treatment is to target neurotoxicity instead of focusing on the blood vessel blockade, or in addition to clot lysis. However, preventing excitotoxicity is difficult because glutamate receptors have a critical role in many brain functions. AMPA/kainate receptor antagonists such as NBQX or MPQX can reduce neurological deficits in animal models of autoimmune damage (Smith et al., 2000), but these drugs are too toxic for clinical use. Other strategies, such as blocking the glycine site of the NMDA receptor for treating ischemic stroke have been ineffective in improving outcomes (Lees et al., 2000; Sacco et al., 2001).

The interactions between ionic glutamate receptors and other proteins such as GluR2-GAPDH and NR2-PSD-95 can improve cell survival after ischemic insults, and thus represent another approach to neuroprotective treatments after stroke (Sattler et al., 1999; Zhai et al., 2013). This strategy is attractive because the basic signal transducing functions of the channels are not blocked as they would be by a conventional antagonist. Thus, a more subtle modulation of ion channel function can be achieved, with the hope of reducing excitotoxicity while sparing normal neurotransmission. Those *in vitro* and animal model experiments used small interfering peptides to disrupt the channel-protein interactions, but these peptides may not be ideal for human clinical use. Therefore, an important priority for future research is the development of suitable drugs targeting the ion channel-protein interactions involved in excitotoxicity.

In summary, ion channel function is modulated by interactions with other proteins. Various proteins influence the number and position of particular subunits in the assembled channel, the dynamics of receptor trafficking and targeting to designated subcellular areas, as well as the assembly, stability, and turnover of the receptor on the membrane and in intracellular signaling pathways. These interactions permit the function of ion channels to be fine-tuned according to both external stimuli and intracellular states. This network of molecular interactions allows for complex signal processing by neurons and also reveals new targets for pharmacological manipulation of neuron function that may be clinically useful. Knowledge of ion channel interactions has progressed rapidly and more such interactions are likely to emerge.

### Conflict of interest statement

The authors declare that the research was conducted in the absence of any commercial or financial relationships that could be construed as a potential conflict of interest.

## References

[B1] Aarts M. Liu Y. Liu L. Besshoh S. Arundine M. Gurd J. W. (2002). Treatment of ischemic brain damage by perturbing NMDA receptor- PSD-95 protein interactions. Science 298, 846–850 10.1126/science.1072873 12399596

[B2] Albensi B. C. (2007). The NMDA receptor/ion channel complex: a drug target for modulating synaptic plasticity and excitotoxicity. Curr. Pharm. Des. 13, 3185–3194 10.2174/138161207782341321 18045168

[B3] Albuquerque E. X. Pereira E. F. Alkondon M. Rogers S. W. (2009). Mammalian nicotinic acetylcholine receptors: from structure to function. Physiol. Rev. 89, 73–120 10.1152/physrev.00015.2008 19126755 PMC2713585

[B4] Allison D. W. Gelfand V. I. Spector I. Craig A. M. (1998). Role of actin in anchoring postsynaptic receptors in cultured hippocampal neurons: differential attachment of NMDA versus AMPA receptors. J. Neurosci. 18, 2423–2436 9502803 10.1523/JNEUROSCI.18-07-02423.1998 PMC6793094

[B5] Anggono V. Huganir R. L. (2012). Regulation of AMPA receptor trafficking and synaptic plasticity. Curr. Opin. Neurobiol. 22, 461–469 10.1016/j.conb.2011.12.006 22217700 PMC3392447

[B6] Ashcroft F. M. (2006). From molecule to malady. Nature 440, 440–447 10.1038/nature04707 16554803

[B7] Chen Z. W. Olsen R. W. (2007). GABAA receptor associated proteins: a key factor regulating GABAA receptor function. J. Neurochem. 100, 279–294 10.1111/j.1471-4159.2006.04206.x 17083446

[B8] Connolly C. N. Wafford K. A. (2004). The Cys-loop superfamily of ligand-gated ion channels: the impact of receptor structure on function. Biochem. Soc. Trans. 32, 529–534 10.1042/BST0320529 15157178

[B9] Cui H. Hayashi A. Sun H. S. Belmares M. P. Cobey C. Phan T. (2007). PDZ protein interactions underlying NMDA receptor-mediated excitotoxicity and neuroprotection by PSD-95 inhibitors. J. Neurosci. 27, 9901–9915 10.1523/JNEUROSCI.1464-07.2007 17855605 PMC6672641

[B10] Dacosta C. J. Baenziger J. E. (2013). Gating of pentameric ligand-gated ion channels: structural insights and ambiguities. Structure 21, 1271–1283 10.1016/j.str.2013.06.019 23931140

[B11] Dent J. A. (2010). The evolution of pentameric ligand-gated ion channels. Adv. Exp. Med. Biol. 683, 11–23 10.1007/978-1-4419-6445-8_2 20737785

[B12] Dingledine R. Borges K. Bowie D. Traynelis S. F. (1999). The glutamate receptor ion channels. Pharmacol. Rev. 51, 7–61 10049997

[B13] Durand G. M. Gregor P. Zheng X. Bennett M. V. Uhl G. R. Zukin R. S. (1992). Cloning of an apparent splice variant of the rat N-methyl-D-aspartate receptor NMDAR1 with altered sensitivity to polyamines and activators of protein kinase C. Proc. Natl. Acad. Sci. U.S.A. 89, 9359–9363 10.1073/pnas.89.19.9359 1409641 PMC50126

[B14] Gotti C. Clementi F. Fornari A. Gaimarri A. Guiducci S. Manfredi I. (2009). Structural and functional diversity of native brain neuronal nicotinic receptors. Biochem. Pharmacol. 78, 703–711 10.1016/j.bcp.2009.05.024 19481063

[B15] Grossman A. W. Broderick J. P. (2013). Advances and challenges in treatment and prevention of ischemic stroke. Ann. Neurol. 74, 363–372 10.1002/ana.23993 23929628

[B16] Hollmann M. Boulter J. Maron C. Beasley L. Sullivan J. Pecht G. (1993). Zinc potentiates agonist-induced currents at certain splice variants of the NMDA receptor. Neuron 10, 943–954 10.1016/0896-6273(93)90209-A 7684237

[B17] Jarvis S. E. Magga J. M. Beedle A. M. Braun J. E. Zamponi G. W. (2000). G protein modulation of N-type calcium channels is facilitated by physical interactions between syntaxin 1A and Gbetagamma. J. Biol. Chem. 275, 6388–6394 10.1074/jbc.275.9.6388 10692440

[B18] Kalia L. V. Pitcher G. M. Pelkey K. A. Salter M. W. (2006). PSD-95 is a negative regulator of the tyrosine kinase Src in the NMDA receptor complex. EMBO J. 25, 4971–4982 10.1038/sj.emboj.7601342 16990796 PMC1618112

[B19] Kisilevsky A. E. Mulligan S. J. Altier C. Iftinca M. C. Varela D. Tai C. (2008). D1 receptors physically interact with N-type calcium channels to regulate channel distribution and dendritic calcium entry. Neuron 58, 557–570 10.1016/j.neuron.2008.03.002 18498737

[B20] Kittler J. T. Moss S. J. (2003). Modulation of GABAA receptor activity by phosphorylation and receptor trafficking: implications for the efficacy of synaptic inhibition. Curr. Opin. Neurobiol. 13, 341–347 10.1016/S0959-4388(03)00064-3 12850219

[B21] Kornau H. C. Schenker L. T. Kennedy M. B. Seeburg P. H. (1995). Domain interaction between NMDA receptor subunits and the postsynaptic density protein PSD-95. Science 269, 1737–1740 10.1126/science.7569905 7569905

[B22] Kozuska J. L. Paulsen I. M. (2012). The Cys-loop pentameric ligand-gated ion channel receptors: 50 years on. Can. J. Physiol. Pharmacol. 90, 771–782 10.1139/y2012-018 22493950

[B23] Lee F. J. Xue S. Pei L. Vukusic B. Chery N. Wang Y. (2002). Dual regulation of NMDA receptor functions by direct protein-protein interactions with the dopamine D1 receptor. Cell 111, 219–230 10.1016/S0092-8674(02)00962-5 12408866

[B24] Lees K. R. Asplund K. Carolei A. Davis S. M. Diener H. C. Kaste M. (2000). Glycine antagonist (gavestinel) in neuroprotection (GAIN International) in patients with acute stroke: a randomised controlled trial. GAIN International Investigators. Lancet 355, 1949–1954 10.1016/S0140-6736(00)02326-6 10859040

[B25] Li K. W. Chen N. Smit A. B. (2013a). Interaction proteomics of the AMPA receptor: towards identification of receptor sub-complexes. Amino Acids 44, 1247–1251 10.1007/s00726-013-1461-9 23344883

[B26] Li S. Li Z. Pei L. Le A. D. Liu F. (2012). The alpha7nACh-NMDA receptor complex is involved in cue-induced reinstatement of nicotine seeking. J. Exp. Med. 209, 2141–2147 10.1084/jem.20121270 23091164 PMC3501362

[B27] Li S. Nai Q. Lipina T. V. Roder J. C. Liu F. (2013b). alpha7nAchR/NMDAR coupling affects NMDAR function and object recognition. Mol. Brain 6:58 10.1186/1756-6606-6-58 24360204 PMC3878138

[B28] Liu F. Wan Q. Pristupa Z. B. Yu X. M. Wang Y. T. Niznik H. B. (2000). Direct protein-protein coupling enables cross-talk between dopamine D5 and gamma-aminobutyric acid A receptors. Nature 403, 274–280 10.1038/35001232 10659839

[B29] Magga J. M. Jarvis S. E. Arnot M. I. Zamponi G. W. Braun J. E. (2000). Cysteine string protein regulates G protein modulation of N-type calcium channels. Neuron 28, 195–204 10.1016/S0896-6273(00)00096-9 11086994

[B30] Maksay G. (2009). Ligand-gated pentameric ion channels, from binding to gating. Curr. Mol. Pharmacol. 2, 253–262 10.2174/1874467210902030253 20021463

[B31] Michaelis E. K. (1998). Molecular biology of glutamate receptors in the central nervous system and their role in excitotoxicity, oxidative stress and aging. Prog. Neurobiol. 54, 369–415 10.1016/S0301-0082(97)00055-5 9522394

[B32] Niethammer M. Kim E. Sheng M. (1996). Interaction between the C terminus of NMDA receptor subunits and multiple members of the PSD-95 family of membrane-associated guanylate kinases. J. Neurosci. 16, 2157–2163 8601796 10.1523/JNEUROSCI.16-07-02157.1996 PMC6578538

[B33] Nishida M. MacKinnon R. (2002). Structural basis of inward rectification: cytoplasmic pore of the G protein-gated inward rectifier GIRK1 at 1.8 A resolution. Cell 111, 957–965 10.1016/S0092-8674(02)01227-8 12507423

[B34] Pei L. Lee F. J. Moszczynska A. Vukusic B. Liu F. (2004). Regulation of dopamine D1 receptor function by physical interaction with the NMDA receptors. J. Neurosci. 24, 1149–1158 10.1523/JNEUROSCI.3922-03.2004 14762133 PMC6793575

[B35] Rojas A. Dingledine R. (2013). Ionotropic glutamate receptors: regulation by G-protein-coupled receptors. Mol. Pharmacol. 83, 746–752 10.1124/mol.112.083352 23348498 PMC6067632

[B36] Sacco R. L. DeRosa J. T. Haley E. C. Jr. Levin B. Ordronneau P. Phillips S. J. (2001). Glycine antagonist in neuroprotection for patients with acute stroke: GAIN Americas: a randomized controlled trial. JAMA 285, 1719–1728 10.1001/jama.285.13.1719 11277826

[B37] Sattler R. Xiong Z. Lu W. Y. Hafner M. MacDonald J. F. Tymianski M. (1999). Specific coupling of NMDA receptor activation to nitric oxide neurotoxicity by PSD-95 protein. Science 284, 1845–1848 10.1126/science.284.5421.1845 10364559

[B38] Smith T. Groom A. Zhu B. Turski L. (2000). Autoimmune encephalomyelitis ameliorated by AMPA antagonists. Nat. Med. 6, 62–66 10.1038/71548 10613825

[B39] Sugihara H. Moriyoshi K. Ishii T. Masu M. Nakanishi S. (1992). Structures and properties of seven isoforms of the NMDA receptor generated by alternative splicing. Biochem. Biophys. Res. Commun. 185, 826–832 10.1016/0006-291X(92)91701-Q 1352681

[B40] Sun H. S. Doucette T. A. Liu Y. Fang Y. Teves L. Aarts M. (2008). Effectiveness of PSD95 inhibitors in permanent and transient focal ischemia in the rat. Stroke 39, 2544–2553 10.1161/STROKEAHA.107.506048 18617669

[B41] Tarze A. Deniaud A. Le Bras M. Maillier E. Molle D. Larochette N. (2007). GAPDH, a novel regulator of the pro-apoptotic mitochondrial membrane permeabilization. Oncogene 26, 2606–2620 10.1038/sj.onc.1210074 17072346

[B42] Wang M. Li S. Zhang H. Pei L. Zou S. Lee F. J. (2012). Direct interaction between GluR2 and GAPDH regulates AMPAR-mediated excitotoxicity. Mol. Brain 5:13 10.1186/1756-6606-5-13 22537872 PMC3407747

[B43] Zamponi G. W. Bourinet E. Nelson D. Nargeot J. Snutch T. P. (1997). Crosstalk between G proteins and protein kinase C mediated by the calcium channel alpha1 subunit. Nature 385, 442–446 10.1038/385442a0 9009192

[B44] Zhai D. Li S. Wang M. Chin K. Liu F. (2013). Disruption of the GluR2/GAPDH complex protects against ischemia-induced neuronal damage. Neurobiol. Dis. 54, 392–403 10.1016/j.nbd.2013.01.013 23360709

[B45] Zou S. Li L. Pei L. Vukusic B. Van Tol H. H. Lee F. J. (2005). Protein-protein coupling/uncoupling enables dopamine D2 receptor regulation of AMPA receptor-mediated excitotoxicity. J. Neurosci. 25, 4385–4395 10.1523/JNEUROSCI.5099-04.2005 15858065 PMC6725121

